# Effects of dietary rumen undegradable protein:rumen degradable protein ratio on nitrogen metabolism in Hanwoo steers

**DOI:** 10.5713/ab.24.0592

**Published:** 2025-02-27

**Authors:** Sang Yeob Kim, Rajaraman Bharanidharan, Seyun Im, Kyoung Hoon Kim, Joonpyo Oh, Hyun Jin Kim, Jaesung Lee, Kamburawala Kankanamge Tharindu Namal Ranaweera, Jin Woo Jeong, Jun Seok Oh, Sang Hyun Lee, Myunggi Baik

**Affiliations:** 1 Department of Agricultural Biotechnology and Research Institute of Agriculture and Life Sciences, College of Agriculture and Life Sciences, Seoul National University, Seoul, Korea; 2 Department of Eco-friendly Livestock Science, Institute of Green Bio Science and Technology, Seoul National University, Pyeongchang, Korea; 3 Cargill Animal Nutrition Korea, Seongnam, Korea

**Keywords:** Dietary Rumen Undegradable Protein, Fermented Heat-Treated Soybean Meal, Korean Cattle Steers, Nitrogen Excretion, Retained Nitrogen

## Abstract

**Objective:**

We investigated the effects of dietary rumen undegradable protein (RUP): rumen degradable protein (RDP) ratio on growth performance, nitrogen (N) metabolism, and rumen and blood parameters in Hanwoo (Korean cattle) steers.

**Methods:**

Eight Hanwoo steers (average body weight, 393 kg) were allocated to two groups and fed with a high RUP:RDP ratio (46.9:53.1 based on crude protein) or a low RUP:RDP ratio (30.6:69.4) concentrate with iso-crude protein content in a 2×2 Latin square design in two successive periods.

**Results:**

The high RUP:RDP group had higher (p<0.01) average daily gain, and lower (p<0.05) ruminal ammonia and plasma urea N concentrations than the low RUP:RDP group. The high RUP:RDP group had lower (p<0.05) urinary N excretion and urinary N per N intake and higher (p<0.1) tendency of retained N than the low RUP:RDP group. The high RUP:RDP group had higher (p<0.1) tendency of N utilization efficiency (retained N per N intake: 28.7% vs. 25.5%) than the low RUP:RDP group. The high RUP:RDP group had a lower (p<0.1) tendency of urinary N per total N excretion, but a higher tendency of fecal N per total N excretion.

**Conclusion:**

A high ratio of dietary RUP:RDP may improve N utilization efficiency by reducing urinary N excretion, which may be beneficial for the environment through reducing atmospheric ammonia emissions.

## INTRODUCTION

In cattle, nitrogen (N) efficiency is generally low, at approximately 24.7% for dairy cattle and 14.3% for beef cattle [1,2]. Ruminants have a more complex N metabolism than monogastric animals due to their unique rumen microbial flora, which allows them to process dietary N. In ruminants overfed with dietary N, excess N is converted to ammonia (NH_3_) by rumen microbes and subsequently excreted in urine primarily as urea [1]. Therefore, it is important to provide adequate amounts of dietary N for the proper growth of rumen microbes as well as for the needs of the host animal to minimize N loss.

Rumen microbes degrade rumen degradable protein (RDP) and subsequently produce mainly peptides, amino acids, and eventually NH_3_ [3]. These compounds are utilized by rumen microbes to synthesize microbial proteins [1]. However, excessive RDP supply results in the production of excess NH_3_, which is absorbed into the bloodstream through the rumen wall, converted to urea in the liver, and then excreted in urine [4]. Thus, excessive RDP can decrease the efficiency of dietary N utilization in ruminants. In contrast, rumen undegradable protein (RUP) is a bypass protein that is not degraded in the rumen, but instead flows into the small intestine where it is digested and absorbed into the blood of ruminant [5]. RUP supplementation moderates ruminal NH_3_ levels [6] and can improve the whole-tract digestion of nutrients by providing a continuous, stable source of recyclable N for ruminants following the prolonged deamination of amino acids [7]. A recent study reported increases in retained N and average daily gain in RUP-supplemented steers [8]. Costa et al [9] also reported that supplying a RUP-enriched diet to beef cows during late gestation improved their performance, and also increased the numbers of muscle fibers and rates of intramuscular adipogenesis in their offspring. Therefore, reducing RDP and increasing RUP appears to reduce ruminal NH_3_ production and improve feed N efficiency. RUP supply also reduced N excretion in lactating Holstein cows, particularly as urinary N [10], which can contaminate the environment more rapidly than fecal N [11].

Previous studies have demonstrated that fermentation of soybean meal improved its nutritional value [12] and animal growth performance through the elimination of anti-nutritional factors [13]. Heat-treating soybean meal can also improve its efficiency as a protein source by reducing ruminal protein degradation [14]. Appropriate heat treatment of soybean protein also promoted amino acid digestibility by inhibiting protease inhibitor activity in ruminants and non-ruminants [15,16]. Chemically or physically treated soybean meal usually has high RUP content and is considered high-quality feed source in terms of amino acid balance [8]. A previous study reported that the *in situ* proportions of RUP in untreated and heat-treated soybean meal were 37.9% and 73.1%, respectively [17]. Partial replacement of soybean meal with heat-treated soybean meal to increase RUP has improved feed intake and milk yield without affecting feed efficiency [18]. Several studies reported that increasing the dietary RUP:RDP ratio improved feed efficiency and apparent N efficiency in lactating Holstein dairy cows [19,20]. Providing a higher dietary RUP:RDP ratio to Holstein dairy calves improved average daily gain, final body weight, and feed efficiency [21]. However, few studies have examined the effects of fermented heat-treated soybean meal on N metabolism in beef cattle. Feeding a high RUP:RDP ratio by using fermented heat-treated soybean meal could increase the efficiency of N utilization and reduce N loss in Hanwoo steers. We investigated the effects of dietary RUP:RDP ratio on growth performance, N metabolism, and rumen and blood parameters in Hanwoo steers.

## MATERIALS AND METHODS

### Ethics statement

Experimental procedures were approved by the Seoul National University Institutional Animal Care and Use Committee (SNUIACUC: SNU-210907-5), Korea and conducted in accordance with its Animal Experiment Guidelines.

### Animals and experimental design

This research was conducted at the Animal Farm of the Pyeongchang Campus of Seoul National University, Korea. A total of eight Hanwoo steers (14.8±0.14 months of age and initial body weight of 392.9±5.61 kg) were used in a replicated 2×2 Latin square design. Animals were assigned to a low RUP:RDP or high RUP:RDP group based on two experimental concentrates in a cross-over trial over two successive periods. Each period included 16 days for feed adaptation at a stanchion in a barn, 1 day for blood and rumen fluid sampling in the barn, 2 days to adapt to a metabolic cage (137 cm wide×256 cm deep×200 cm high, with a rubber mat covering the floor), and 5 days for fecal and urine sampling in the cage. Each period was separated by a 2-day washout period in the barn. Thus, the total feeding period was 50 days.

### Experimental diet and feeding trial

Experimental concentrates (low RUP:RDP and high RUP: RDP) were provided in pellet form by Cargill Agri Purina, Inc. (Seongnam, Korea). Table 1 lists the ingredients of concentrate pellets: each type contained the same 18% dry matter (DM) crude protein (CP) content, while the high RUP:RDP ratio (46.9:53.1 based on CP) concentrate had 16.3% higher RUP level than the low RUP:RDP ratio (30.6:69.4) concentrate. The 3.0% DM of fermented heat-treated soybean meal (SoELAB-PASS; FEEDUP Co., Ltd., Nonsan, Korea) was used as the RUP source for the high RUP concentrate. The ingredients and chemical composition of the diets are shown in Table 2.

During the feeding trial, both groups were fed either low RUP:RDP or high RUP:RDP concentrate (approximately 1.59% of their body weight per day per head), together with rice straw (2.5 kg per day per head). Rice straw was purchased from Daehwa Nonghyup (Pyeongchang, Korea). The rice straw was divided into two equal portions and supplied at 8:00 and 14:00. The residual forage diet was weighed 2 h after feeding. During feeding in the barn (days 1 to 17 and 25 to 43), concentrates were provided by the DeLaval Delpro automatic feeding station (DeLaval, Tumba, Sweden). Concentrate intake was automatically recorded on a Delpro system computer. Feeding in the metabolic cages (days 18 to 24 and 44 to 50) followed the same schedule as feeding in the barn, with concentrates supplied 1 h after forage supply. The residual diet was weighed before the next feeding. Fresh water was freely available via automatic drinkers. Feed samples were collected at 2-week intervals and stored at −70°C until analysis. Body weight was measured at 8:00 before feeding on the first and last days of the experiment in each period.

### Total urine and fecal sample collection

Total urine and fecal samples were collected in individual metabolic cages equipped with a feeder and water bowl. All feces were collected every 2 h and weighed once per day at 9:00 for 5 days, and 10% of the total feces were frozen daily at −70°C until analysis. Total urine was collected via a rubber funnel connected by rubber tubing to a 50-L plastic barrel containing 300 mL of 4N H_2_SO_4_ to prevent N loss. Urine was weighed daily and 5% of the total urine was frozen daily at −70°C until analysis.

### Chemical analysis and digestibility calculation

Fecal samples were dried in an oven at 65°C for 72 h, and moisture content was estimated. The DM (method 930.15), CP (Kjeldahl N×6.25; method 981.10), ether extract (method 920.39), and ash (method 942.05) contents of diets and feces, as well as the urine N content (method 955.04), were determined using analytical methods provided by the Association of Official Analytical Chemists (AOAC) [22]. The neutral detergent fiber and acid detergent fiber contents of diets and feces were analyzed using a sequential method in an ANKOM 200 fiber analyzer (Ankom Technology, Macedon, NY, USA). Details of the method are given in Van Soest et al [23].

Digestibility was calculated as described by Montoya and Leterme [24]:


$$\text{Apparent digestibility }(\%\;\text{of DM})=\left(\frac{\text{Ingested nutrient}-\text{Excreted nutrient}}{\text{Ingested nutrient}}\right)\times 100$$

For amino acid analysis, frozen ground concentrate was taken in vials and 6N HCl was added. The vials were sealed and were kept at 130°C for 24 h for complete protein hydrolysis. The vial contents were filtered through a syringe filter (pore size, 0.45 μm), and the filtered samples were neutralized and diluted with triple-distilled water. Next, 1 μL of the contents were dissolved in 5 μL borate buffer (5061-3339; Agilent Technologies, Santa Clara, CA, USA), and then 1 μL o-phthaldialdehyde (OPA) reagent (5061-3335; Agilent Technologies) and 1 μL fluorenyl methoxycarbonyl (FMOC) solution (5061-3337; Agilent Technologies) were added, together with 32 μL distilled water for pre-column derivation. High-performance liquid chromatography (HPLC) analysis was performed using a Dionex Ultimate 3000 (Thermo Scientific, Waltham, MA, USA) with an Agilent 1260 Infinity Fluorescence Detector (Agilent Technologies) at emission and excitation wavelengths of 450 and 340 nm, respectively, for OPA and 305 and 266 nm, respectively, for FMOC. Free amino acid separation was performed using an Inno C18 column (4.6 mm×150 mm, 5 μm; Youngjin Biochrom Co., Seoul, Korea). Detection was performed simultaneously using an ultraviolet (UV) light detector at 338 nm, with the column temperature set at 40°C, and the injection volume set at 1 μL. Mobile phase A was sodium phosphate (40 mM, pH 7), and mobile phase B was distilled water, acetonitrile, and methanol (10:45:45 v/v %), at a flow rate of 1.5 mL min^−1^.

### Blood collection and analysis

Blood samples were collected three times: at morning fasting (0 h), and at 3 and 6 h after morning feeding on the day before the metabolic cage adaptation date in each period. Blood samples were collected via jugular venipuncture into ethylenediaminetetraacetic acid-treated vacutainers (20 mL). Plasma was separated by centrifugation at 2,500×g for 15 min at 4°C and stored at 70°C until analysis.

Plasma metabolites were analyzed using a fully automated Cobas 6000 C501 analyzer (Roche Diagnostics, Mannheim, Germany) with specific kits. Plasma albumin was analyzed via a colorimetric method using the Albumin Gen.2 kit (Roche Diagnostics), blood urea nitrogen (BUN) was analyzed via kinetic/photometric methods using the Urea/BUN kit (Roche Diagnostics), glucose was analyzed via enzymatic UV and hexokinase methods using the Glucose HK kit (Roche Diagnostics), total protein was analyzed via a colorimetric method using the Total Protein Gen.2 kit (Roche Diagnostics), and free fatty acid was analyzed via a colorimetric method using the non-esterified fatty acid HR.II kit (Wako Pure Chemical Industries Ltd., Osaka, Japan).

Plasma samples were mixed with buffer solution (0.1 M perchloric acid+0.1% meta phosphoric acid+distilled water), extracted by a sonicator for 1 h, shaken at room temperature for 1 h, and then filtered through a hydrophilic syringe filter (pore size, 0.2 μm). The free amino acid levels of the filtered plasma samples were then analyzed by HPLC as described above for concentrate analysis.

### Rumen fluid collection and analysis

Rumen fluid samples were collected twice, at 1 and 3 h after morning feeding on the day before the metabolic cage adaptation date of each period. Rumen fluid was collected using the oral stomach tube method described by Shen et al [25], and filtered through four layers of cheesecloth. The pH was immediately measured using a portable pH meter (Ohaus Corp., Parsippany, NJ, USA) and the rumen fluid was stored at −70°C until analysis.

Frozen rumen fluid was thawed and a 1-mL sample was mixed with 0.1 mL of 50% metaphosphoric acid for volatile fatty acid (VFA) analysis. VFA concentrations were determined using an Agilent Tech 7890B gas chromatography system (Agilent Technologies) with a flame ionization detector, as previously used in our laboratory [26]. The NH_3_-N content of the rumen fluid was determined utilizing a UV spectrophotometer (SpectraMax iD3; Molecular Devices, San Jose, CA, USA) and the modified colorimetric method described by Chaney and Marbach [27].

For microbial population analysis, 1-h rumen fluid samples were immediately frozen in liquid nitrogen upon collection and stored at −70°C until analysis. The method used for microbial analysis has been described in detail by Jeong et al [28]. Briefly, frozen rumen fluid was thawed and centrifuged at 13,000×g and 4°C for 30 min. Pellet samples were ground with liquid nitrogen, and the powder was used to determine the microbial population by genomic DNA extraction and quantitative real-time polymerase chain reaction. Supplement 1 lists the primers and polymerase chain reaction conditions.

### Statistical analyses

All data were analyzed as a 2×2 Latin square design with duplication using the PROC MIXED procedure in SAS v9.4 (SAS Institute Inc., Cary, NC, USA). Each animal was considered as an experimental unit. The model included dietary treatment as a fixed effect, with animal nested within diet group as a random effect. Several variance–covariance structures (compound symmetry, autoregressive type 1, and Toeplitz) were tested, and the structure that minimized the Schwarz’s Bayesian information criterion was selected. Means were calculated using the LSMEANS statement, and data were expressed as least squares means and standard error of the mean. Statistical significance was evaluated at p≤0.05, and a tendency was determined at 0.05<p≤0.10.

## RESULTS

### Growth performance

Average daily gain was higher (p = 0.001) in the high RUP: RDP group than in the low RUP:RDP group. Feed intake and the digestibility of DM, organic matter, CP, neutral detergent fiber, and acid detergent fiber were not affected (p≥0.11) by RUP:RDP ratio. Both intake (p<0.001) and digestibility (p = 0.038) of the ether extract were higher in the high RUP:RDP group than in the low RUP:RDP group (Table 3).

### Rumen fermentation parameters and microbial population

Rumen pH and total VFA were not affected (p≥0.20) by RUP:RDP ratio (Table 4). However, rumen NH_3_ was lower (p≤0.023) in the high RUP:RDP group than in the low RUP:RDP group at 1 and 3 h after feeding. The rumen acetate proportion was lower (p≤0.005) in the high RUP:RDP group than in the low RUP:RDP group at 1 and 3 h, whereas the propionate proportion was higher (p≤0.03). The rumen iso-butyrate proportion was lower (p = 0.029) in the high RUP:RDP group than in the low RUP:RDP group at 1 h, and tended to be lower (p = 0.056) at 3 h.

The acetate:propionate ratio was lower (p = 0.042) in the high RUP:RDP group than in the low RUP:RDP group at 1 h and tended to be lower (p = 0.076) at 3 h. Butyrate, iso-butyrate, and valerate proportions were not affected (p≥0.16) by RUP:RDP ratio (Table 4).

**Figure 1:**
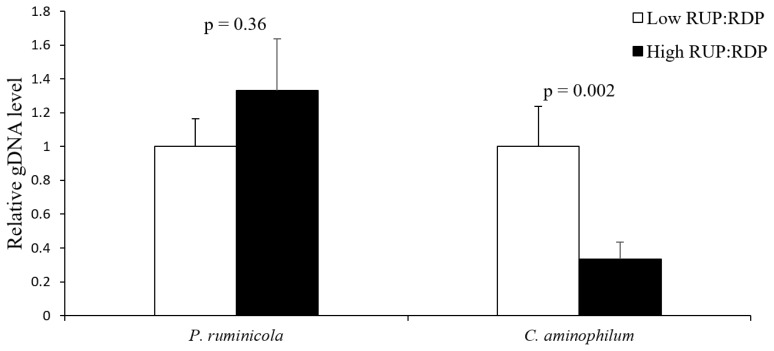
Effects of dietary rumen undegradable protein (RUP):rumen degradable protein (RDP) ratio on the rumen bacterial population in Hanwoo steers. The bacterial population was measured by quantitative real-time polymerase chain reaction, using the total bacterial 16S rRNA gene as the reference gene. Relative genomic DNA (gDNA) levels of the low RUP:RDP group for each bacterium were normalized to 1.0-fold. Values are fold differences+standard error. *P. ruminicola*, *Prevotella ruminicola*; *C*. *aminophilum*, *Clostridium aminophilum*.

Ruminal *Prevotella ruminicola* abundance was not affected (p = 0.36) by RUP:RDP ratio (Figure 1). The relative levels of *Clostridium aminophilum* were lower (p = 0.002) in the high RUP:RDP group than in the low RUP:RDP group (Figure 1).

### Blood parameters

BUN was lower (p≤0.047) in the high RUP:RDP group than in the low RUP:RDP group at fasting (0 h) and 3 and 6 h after feeding (Table 5). Blood non-esterified fatty acid tended to be lower (p = 0.082) in the high RUP:RDP group than in the low RUP:RDP group at 3 h. Blood albumin, glucose, and total protein concentrations were not affected (p≥0.17) by RUP: RDP ratio (Table 5).

### Plasma amino acid concentrations

Plasma leucine levels were higher (p = 0.046) in the high RUP:RDP group than in the low RUP:RDP group at 0 h and tended to be higher (p = 0.091) at 3 h (Table 6). Plasma valine (p = 0.092) and aspartic acid levels (p = 0.082) tended to be higher in the high RUP:RDP group than in the low RUP:RDP group at 0 h. Glycine levels were higher (p = 0.01) in the high RUP:RDP group than in the low RUP:RDP group at 3 h. All other amino acids were unaffected (p≥0.17) by RUP:RDP ratio (Table 6).

### N metabolism

N intake and fecal and urinary excretion levels were not affected (p≥0.34) by RUP:RDP ratio (Table 7). Fecal N excretion (g/day) and fecal N percentage of total N excreted were not affected (p≥0.18) by RUP:RDP ratio. Both urinary N excretion (g/day) (p = 0.02) and the urinary N percentage of N intake (p = 0.029) were lower in the high RUP:RDP group than in the low RUP:RDP group. Both total (fecal plus urinary) N excretion (g/day) (p = 0.10) and the total N excretion percentage of N intake (p = 0.095) tended to be lower in the high RUP:RDP group than in the low RUP:RDP group. Both retained N (g/day) (p = 0.097) and the retained N percentage of N intake (p = 0.095) tended to be higher in the high RUP:RDP group than in the low RUP:RDP group. The urinary N percentage of total N excretion tended to be lower (p = 0.055) in the high RUP:RDP group than in the low RUP:RDP group, whereas the fecal N percentage of total N excretion tended to be higher (p = 0.055) (Table 7).

## DISCUSSION

In this study, we found a greater average daily gain in the high RUP:RDP group compared to the low RUP:RDP group. Similarly, RUP supplementation in Nellore steers increased average daily gain and retained N [8]. These results suggest that a high RUP:RDP diet may contribute to increased N utilization efficiency, resulting in greater body weight gain. In the present study, the average daily gain was 0.39 and 0.68 kg/day in the low RUP:RDP group and high RUP:RDP group, respectively. A previous study using Hanwoo steers of similar age and body weight to the present study reported an average daily gain of 0.57 to 0.83 kg/day [26]. The slightly lower average daily gain in the current study may be related to retarded growth caused by the stress and cramped space of metabolic cage rearing.

We found higher ether extract digestibility in the high RUP:RDP group compared to the low RUP:RDP group. In this study, the high RUP:RDP concentrate contained 5% higher levels of corn distillers-dried grains with solubles (DDGS) than the low RUP:RDP concentrate. In a previous study, Holstein cows fed 18% DDGS along with 13.2% ether extract showed higher digestibility of the ether extract than those fed no DDGS [29]. Therefore, it is possible that the higher DDGS content in the high RUP:RDP concentrate may have partially contributed to the increased ether extract digestibility in this study.

Our results of VFA profile were consistent with a previous study that showed the decreased acetate to propionate ratio when soybean meal was replaced with fermented soybean meal in Holstein cattle [30].

Similarly, the inclusion of DDGS resulted in lower ruminal acetate proportions and higher propionate proportions in Holstein cattle [29,31]. Taken together, these results suggest that fermented heat-treated soybean meal or higher corn DDGS levels in the high RUP:RDP diet may have contributed to these VFA profiles. Our study showed a lower ruminal iso-butyrate content in the high RUP:RDP group compared to the low RUP:RDP group. Iso-butyrate is one of the branched short-chain fatty acid produced by microbes during the fermentation of valine [32] and the present study showed no difference in plasma valine concentration between two groups. A previous study reported that iso-butyrate was not affected by dietary RUP levels in Holstein cows [10], so our results remain unexplained.

Previous research has shown that decreased levels of ruminal NH_3_ generally decrease N transport to the blood, thereby limiting blood urea N concentrations [8]. In our study, both ruminal NH_3_ and plasma urea N concentrations were lower in the high RUP:RDP group compared to the low RUP:RDP group. The dietary urea contained in the low RUP:RDP diet may also contribute partially to the higher ruminal NH_3_ levels in the low RUP:RDP animal than in the high RUP:RDP animal [33]. The population of *Clostridium aminophilum*, one of the NH_3_-producing bacteria [34], was also lower in the high RUP:RDP group than in the low RUP:RDP group. Meanwhile, the RUP:RDP ratio did not affect the population of *Prevotella ruminicola*. *Prevotella ruminicola* is known to be a saccharolytic bacteria [35].

In this study, increased tendency of N efficiency in the high RUP:RDP group may contribute in part to the increased body weight gain. In the current study, ruminal NH_3_ and blood urea N concentrations were reduced in the high RUP:RDP group. Previous studies have reported that increased ruminal NH_3_ concentrations result in increased blood urea N levels, thereby increasing urinary N excretion [36–38]. Our study suggests that increased dietary RUP:RDP ratio tend to enhance N utilization efficiency and reduce urinary N excretion. A previous study found that increasing dietary RUP levels decreased urinary N excretion in Holstein cows, but found no differences in fecal N excretion [10]. Urinary N is primarily composed of urea, which is easily degraded into NH_3_ and utilized as a precursor of N_2_O in the soil and atmosphere, such that reducing urinary N excretion in cattle is critical for reducing NH_3_ and N_2_O emissions [1,39,40]. N_2_O is a potent greenhouse gas with a global warming potential approximately 296 times greater than that of carbon dioxide [41]. Thus, reducing urinary N excretion by increasing the dietary RUP:RDP ratio without changing dietary N level could reduce soil and air pollution resulting from N emission and contribute to environmental-friendly livestock production.

## CONCLUSION

Feeding a high RUP:RDP concentrate diet improved body weight gain, reduced ruminal NH_3_ and plasma urea N concentrations, and urinary N excretion compared with a low RUP:RDP concentrate diet in Hanwoo steers, resulting in a higher tendency of retained N. The increased tendency of N utilization efficiency with reduced urinary N excretion may contribute to environmental-friendly livestock production by reducing pollution derived from N emissions.

## Figures and Tables

**Table 1:** Ingredients of the experimental concentrates (% of dry matter)

Ingredient	Low RUP:RDP concentrate	High RUP:RDP concentrate
Flaked corn	22.0	27.0
Corn gluten feed	19.0	7.00
Fine wheat	17.4	15.0
Corn DDGS	12.0	17.0
Palm kernel meal	8.40	20.0
Ground alfalfa pellet	7.00	0.00
Molasses	4.50	5.00
Soy hulls	2.80	0.00
Limestone	2.30	2.50
Fine corn	1.10	1.10
Rice bran	1.00	1.00
Mineral premix^1)^	0.85	0.85
Urea	0.60	0.00
Extruded linseed	0.50	0.00
Glycerin	0.30	0.30
Salt	0.20	0.20
Vitamin premix^1)^	0.05	0.05
SoELAB_PASS^2)^	0.00	3.00
Total	100	100

1) Vitamin and mineral premix contained 1,050 IU vitamin E, 530,000 IU vitamin D3, 2,650,000 IU vitamin A, 10 g Niacin, 13.2 g Fe, 4.4 g Zn, 4.4 g Mn, 2.2 g Cu, 0.44 g Co, and 0.44 g I and per kg of additive (Grobic-DC; Bayer Health Care, Leverkusen, Germany).

2) Source of SoELAB_PASS: FEEDUP Co., Ltd., Nonsan, Korea.

RUP, rumen undegradable protein; RDP, rumen degradable protein; DDGS, distillers dried grains with solubles.

**Table 2:** Ingredients and chemical composition experimental diets and individual feeds

Item	Dietary treatment		Concentrate		Forage
		
	Low RUP:RDP	High RUP:RDP	Low RUP:RDP	High RUP:RDP	Rice straw
Ingredients (% dry matter [DM])	100	100			
Concentrate^1)^	76.6	77.8			
Rice straw	23.4	22.2			
Chemical Composition (% DM)					
DM	88.9	88.7	88.3	88.0	91.0
Crude protein (CP)	14.7	14.9	18.1	18.2	3.61
Ether extract (EE)	2.72	3.05	3.13	3.51	1.40
Ash	8.81	7.78	8.02	6.76	11.4
Crude fiber (CF)	13.1	12.5	6.79	6.40	33.7
Neutral detergent fiber (NDF)	35.4	34.8	25.9	25.7	66.6
Acid detergent fiber (ADF)	17.0	18.1	10.2	12.0	39.6
Nitrogen free extract (NFE)^2)^	60.7	61.8	63.9	65.1	49.9
Non-fiber carbohydrates (NFC)^3)^	38.3	39.4	44.8	45.8	17.0
Rumen undegradable protein^4)^ (RUP, %)	4.48	6.85	5.55	8.52	0.97
RUP^4)^ (% of CP)	29.8	42.5	30.6	46.9	27.0
Rumen degradable protein^4)^ (RDP, %)	10.2	8.10	12.6	9.66	2.63
RDP^4)^ (% of CP)	70.2	57.5	69.4	53.1	73.0
RUP:RDP (% of CP)	0.42	0.74	0.44	0.88	0.37
Metabolizable protein^4)^	9.03	10.6	10.5	12.4	4.28
Essential amino acids (g/100 g)					
Arginine	0.61	0.69	0.74	0.83	0.18
Histidine	0.26	0.24	0.32	0.30	0.04
Isoleucine	0.35	0.39	0.40	0.45	0.17
Leucine	0.85	0.97	1.02	1.16	0.29
Lysine	0.25	0.26	0.28	0.29	0.13
Methionine	0.16	0.17	0.19	0.20	0.05
Phenylalanine	0.44	0.50	0.51	0.59	0.20
Threonine	0.38	0.39	0.44	0.45	0.20
Valine	0.50	0.52	0.57	0.59	0.26
Nonessential amino acids (g/100 g)					
Alanine	0.66	0.65	0.78	0.76	0.26
Aspartic acid	0.66	0.76	0.75	0.88	0.36
Glutamic acid	2.09	2.30	2.58	2.81	0.51
Glycine	0.45	0.44	0.52	0.51	0.21
Proline	0.59	0.62	0.70	0.73	0.21
Serine	0.47	0.52	0.56	0.61	0.20
Tyrosine	0.27	0.29	0.31	0.33	0.15
Total essential amino acids (g/100 g)	3.78	4.12	4.47	4.86	1.52
Total nonessential amino acids (g/100 g)	5.19	5.58	6.20	6.63	1.90
Total amino acids (g/100 g)	8.97	9.70	10.7	11.5	3.42
Energy values					
Total digestible nutrient^4)^ (TDN, %)	67.4	67.8	74.0	74.0	45.9
Digestible energy^5)^ (DE)	2.97	2.99	3.26	3.26	2.02
Metabolizable energy^6)^ (ME)	2.44	2.45	2.68	2.68	1.66

1) Ingredients of concentrate were presented in Table 1.

2) NFE = 100−(CP+EE+Ash+CF).

3) NFC = 100−(CP+EE+Ash+NDF).

4) RUP, RDP, metabolizable protein, and TDN values of concentrates were provided from Cargill Agri Purina, Inc. feed company (Seongnam, Korea), and metabolizable protein of rice straw was also provided from Cargill. RUP, RDP, TDN, and amino acid values of rice straw were obtained from standard table of feed composition in Korea [42].

5) DE = 0.04409×TDN (%) [43].

6) ME = 0.82×DE [43].

**Table 3:** Effects of dietary rumen undegradable protein (RUP):rumen degradable protein (RDP) ratio on feed intake, average daily gain, nutrient intake and digestibility in Hanwoo steers

Item	Low RUP:RDP	High RUP:RDP	SEM	p-value
Feed intake (kg DM/d)				
Total dry matter (DM) intake	6.83	6.73	0.08	0.46
Concentrate DM intake	5.19	5.18	0.05	0.88
Forage DM intake	1.64	1.56	0.06	0.44
Nutrient intake (kg DM/d)				
Organic matter (OM)	6.29	6.26	0.07	0.82
Crude protein (CP)	1.00	1.00	0.01	0.77
Ether extract (EE)	0.16	0.18	0.00	<0.001
Neutral detergent fiber (NDF)	2.18	2.12	0.04	0.38
Acid detergent fiber (ADF)	1.05	1.11	0.02	0.24
Average daily gain (kg/d)	0.39	0.68	0.06	0.0046
Feed efficiency (kg/DM kg)	0.058	0.10	0.01	0.0046
Digestibility (% of DM)				
DM	69.0	69.9	0.79	0.53
OM	73.0	74.3	0.75	0.34
CP	70.8	68.4	0.84	0.18
EE	79.8	83.1	1.05	0.038
NDF	52.5	56.7	1.25	0.11
ADF	50.5	52.6	1.14	0.40

SEM, standard error of the mean.

**Table 4:** Effects of dietary rumen undegradable protein (RUP):rumen degradable protein (RDP) ratio on rumen fermentation profiles in Hanwoo steers

Item	Time after feeding (h)	Low RUP:RDP	High RUP:RDP	SEM	p-value
pH	1	6.49	6.45	0.10	0.86
	3	6.49	6.56	0.07	0.61
Ammonia (mg/dL)	1	26.1	14.9	2.44	0.016
	3	20.0	10.4	2.19	0.023
Total volatile fatty acids (mM)	1	116	101	6.24	0.24
	3	114	96.9	6.56	0.20
Acetate (mol/100 mol)	1	61.1	57.2	1.23	0.0046
	3	62.4	57.8	1.49	0.0031
Propionate (mol/100 mol)	1	20.1	24.8	1.41	0.021
	3	18.4	23.3	1.50	0.030
Iso-butyrate (mol/100 mol)	1	0.85	0.63	0.05	0.029
	3	0.75	0.59	0.04	0.056
Butyrate (mol/100 mol)	1	15.0	14.0	0.60	0.31
	3	15.7	14.9	0.64	0.51
Iso-valerate (mol/100 mol)	1	1.30	1.30	0.09	0.99
	3	1.06	1.35	0.11	0.23
Valerate (mol/100 mol)	1	1.66	2.01	0.13	0.16
	3	1.68	2.03	0.15	0.20
Acetate:propionate	1	3.07	2.53	0.18	0.042
	3	3.43	2.82	0.23	0.076

SEM, standard error of the mean.

**Table 5:** Effects of dietary rumen undegradable protein (RUP):rumen degradable protein (RDP) ratio on plasma parameters in Hanwoo steers

Item	Time after feeding (h)	Low RUP:RDP	High RUP:RDP	SEM	p-value
Blood urea nitrogen (mg/dL)	0	12.4	10.6	0.55	0.047
	3	16.4	13.8	0.58	0.0027
	6	15.0	12.5	0.52	0.012
Albumin (g/dL)	0	3.79	3.69	0.05	0.17
	3	3.78	3.76	0.04	0.83
	6	3.79	3.79	0.06	1
Glucose (mg/dL)	0	84.6	85.0	1.17	0.85
	3	85.0	85.8	1.61	0.64
	6	90.3	90.0	1.42	0.91
Total protein (g/dL)	0	6.56	6.51	0.07	0.70
	3	6.53	6.64	0.06	0.30
	6	6.59	6.76	0.10	0.24
Free fatty acid (μEq/L)	0	115	95.0	15.19	0.42
	3	86.6	63.5	6.39	0.082
	6	103	92.0	13.72	0.66

SEM, standard error of the mean.

**Table 6:** Effects of dietary rumen undegradable protein (RUP):rumen degradable protein (RDP) ratio on plasma amino acids concentrations in Hanwoo steers (mg/L)

Item	Time after feeding (h)	Low RUP:RDP	High RUP:RDP	SEM	p-value
Essential amino acids					
Arginine	0	17.0	19.3	1.08	0.18
	3	15.9	15.9	0.38	0.98
	6	17.0	18.2	0.77	0.51
Histidine	0	11.6	12.9	0.81	0.39
	3	11.8	12.1	0.54	0.74
	6	11.8	12.5	0.51	0.50
Isoleucine	0	13.4	15.3	0.96	0.23
	3	11.5	12.1	0.61	0.57
	6	12.9	13.9	0.75	0.54
Leucine	0	20.3	25.6	1.67	0.046
	3	17.2	21.4	1.31	0.091
	6	19.1	23.6	1.43	0.12
Lysine	0	17.8	16.7	1.16	0.44
	3	12.8	11.8	0.62	0.28
	6	15.1	13.5	0.94	0.41
Methionine	0	4.07	4.32	0.30	0.52
	3	3.77	3.55	0.18	0.29
	6	3.84	3.94	0.21	0.83
Phenylalanine	0	8.59	10.1	0.56	0.16
	3	8.35	8.76	0.37	0.62
	6	8.65	9.48	0.35	0.26
Threonine	0	9.15	9.91	0.78	0.47
	3	7.64	7.93	0.51	0.71
	6	8.51	8.79	0.58	0.83
Tryptophane	0	7.52	8.49	0.62	0.26
	3	7.10	6.99	0.44	0.87
	6	8.09	8.49	0.51	0.72
Valine	0	26.8	33.0	2.28	0.092
	3	25.1	28.6	1.74	0.32
	6	27.0	30.9	1.70	0.26
Nonessential amino acids					
Alanine	0	23.4	22.9	1.31	0.85
	3	20.6	19.4	0.65	0.36
	6	21.9	20.7	0.57	0.29
Asparagine	0	4.38	4.73	0.36	0.45
	3	3.21	3.44	0.22	0.54
	6	3.73	3.99	0.24	0.61
Aspartic acid	0	0.88	1.24	0.10	0.082
	3	0.97	0.94	0.09	0.80
	6	1.01	1.02	0.09	0.90
Glutamic acid	0	8.88	10.7	0.74	0.21
	3	10.2	9.99	0.63	0.79
	6	10.2	10.1	0.53	0.86
Glutamine	0	42.3	43.7	2.75	0.74
	3	44.0	41.3	1.63	0.29
	6	46.5	45.7	1.43	0.81
Glycine	0	24.5	25.2	0.83	0.56
	3	20.1	23.4	0.70	0.010
	6	24.6	25.4	0.83	0.67
Proline	0	6.00	6.36	0.23	0.47
	3	4.80	5.16	0.18	0.35
	6	5.05	5.67	0.26	0.27
Serine	0	8.34	9.29	0.53	0.28
	3	7.38	8.01	0.37	0.32
	6	8.15	8.50	0.38	0.66
Tyrosine	0	11.6	13.7	0.87	0.17
	3	10.7	11.1	0.45	0.67
	6	11.3	12.5	0.62	0.36
Total essential amino acids	0	136	155	9.55	0.19
	3	121	129	5.89	0.46
	6	132	143	7.05	0.46
Total nonessential amino acids	0	130	138	6.73	0.53
	3	122	123	3.54	0.92
	6	132	134	3.42	0.87
Total amino acids	0	267	293	15.9	0.31
	3	243	252	8.82	0.61
	6	264	277	9.82	0.56

SEM, standard error of the mean.

**Table 7:** Effects of dietary rumen undegradable protein (RUP):rumen degradable protein (RDP) ratio on nitrogen (N) balance in Hanwoo steers

Item	Low RUP:RDP	High RUP:RDP	SEM	p-value
N intake (g DM/d)	160	160	1.64	0.77
Fecal excretion (kg DM/d)	2.12	2.03	0.07	0.43
Urine excretion (kg/d)	7.26	5.98	0.63	0.34
N excretion (g/d)	119	114	2.21	0.10
Feces	46.8	50.5	1.51	0.22
Urine	72.2	63.3	2.45	0.02
Retained N^1)^ (g/d)	40.9	45.8	2.42	0.097
N excretion (% of N intake)	74.5	71.3	1.43	0.095
Feces	29.2	31.6	0.84	0.18
Urine	45.3	39.7	1.68	0.029
Retained N^2)^ (% of N intake)	25.5	28.7	1.43	0.095
Proportion of total N excretion (%)				
Fecal N	39.8	45.0	1.49	0.055
Urinary N	60.2	55.0	1.49	0.055

1) Retained N (g/d) = N intake−total N excretion.

2) Retained N (% of N intake) = retained N/N intake×100.

SEM, standard error of the mean; DM, dry matter.

## SUPPLEMENTARY MATERIAL

**Supplement 1**. Primers and gDNA concentrations used for real-time polymerase chain reaction quantification of rumen microbes
