# Supplementary material for: Effects of dietary rumen undegradable protein:rumen degradable protein ratio on nitrogen metabolism in Hanwoo steers
Source: Anim Biosci. 2025 Feb 27;38(6):1182–93. doi: 10.5713/ab.24.0592 (PMC12061585; doi:10.5713/ab.24.0592)
Supplement: Supplementary file 1 [file ab-24-0592-Supplementary-1.pdf]

Supplement 1. Primers and gDNA concentrations used for real-time polymerase chain reaction quantification of rumen microbes

Microbes	gDNA (ng/μL)	Primer concentration	Sequences (5'→3') [1]	Amplicon length (bp)	References
Total bacteria	1	0.3uM<br>0.3uM	F: CGGCAACGAGCGCAACCC<br>R: CCATTGTAGCACGTGTGTAGCC	130	Denman and McSweeney, 2006, FEMS Microbiol. Ecol. 58:572-82.
Prevotella ruminicola	1	0.5uM<br>0.5uM	F: GCGAAAGTCGGATTAATGCTCTATG<br>R: CCCATCCTATAGCGGTAAACCTTTG	78	Khafipour et al., 2009, Appl. Environ. Microbiol. 75:7115-24.
Clostridium aminophilum	10	0.5uM<br>0.5uM	F: ACGGAAATTACAGAAGGAAG<br>R: GTTTCCAAAGCAATTCCAC	560	Patra and Yu, 2014, Appl. Microbiol Biotechnol. 98:897-905.

[1]F = forward; R = reverse.
